# Supplementary material for: Thalamo-cortical spiking model of incremental learning combining perception, context and NREM-sleep
Source: PLoS Comput Biol. 2021 Jun 28;17(6):e1009045. doi: 10.1371/journal.pcbi.1009045 (PMC8270441; doi:10.1371/journal.pcbi.1009045)
Supplement: S1 Text — The reader can find in this section details about the mechanisms in action in ThaCo during the Training and Classification phases (see the ‘Training and classification phases’ and ‘Balanced mini-batch training’ paragraphs, specifically Fig A providing a comparison of incremental learning protocols) and details concerning the implementation and effects of deep sleep dynamics (see ‘Sleep-like oscillatory dynamics’). The specific form of STDP plasticity is described in paragraph ‘Spike-Timing-Dependent Plasticity’ (and depicted in Fig B showing the effects of deep-sleep on network performances) and the neuronal model in ‘The neuron model’. Details about the handwritten digits datatsets are presented in the ‘The datasets of handwritten characters’ paragraph, while paragraph ‘Thalamic coding of visual stimuli’ describes the fuzzy-logic-inspired pre-processing algorithm, adopted for a tuning of thalamic activity that preserves a notion of distance among visual features. The ‘Salt-and-pepper noise’ paragraph is about the method used to add noise to images during training and classification. Finally, the set of parameters needed to configure the spiking model is provided in Table A, paragraph ‘Parameters of the spiking model’. (PDF) [file pcbi.1009045.s001.pdf]

1 Supporting Information

The reader can find in this section details about the mechanisms in action in ThaCo during the Training and Classification phases (see the 'Training and classification phases' and 'Balanced mini-batch training' paragraphs) and details concerning the implementation and effects of deep sleep dynamics (see 'Sleep-like oscillatory dynamics'). The specific form of STDP plasticity is described in paragraph 'Spike-Timing-Dependent Plasticity' and the neuronal model in 'The neuron model'. Details about the handwritten digits datatsets are presented in the 'The datasets of handwritten characters' paragraph, while paragraph 'Thalamic coding of visual stimuli' describes the fuzzy-logic-inspired pre-processing algorithm, adopted for a tuning of thalamic activity that preserves a notion of distance among visual features. The 'Salt-and-pepper noise' paragraph is about the method used to add noise to images during training and classification. Finally, the set of parameters needed to configure the spiking model is provided in Table A, paragraph 'Parameters of the spiking model'.

Training and classification phases Training and classification protocols are depicted in Figs 7A and 7B. During the training phase, the *sensorial perception* (Fig 7A) of each training example is encoded into the thalamus through the activation of a subset of *thalamic* neurons (th), according to the image encoding described in the following ' Thalamic coding of visual stimuli' paragraph. A *time-specific* contextual signal, given in the form of independent Poisson spike trains, rises the perceptual threshold of a subgroup of 20 *cortical* neurons (cx, associated to the specific example) and a *class-specific* contextual signal rises the threshold of 20 *readout* neurons (ro, the digit class to which the example belongs). The perceptual signal drives the enhancement of both: 1) the synapses connecting the active subset of thalamic neurons (coding for the features of the image) with the example-specific group of cortical neurons; 2) synapses connecting the example specific cortical group with the readout group (coding for the correct digit class). During the classification phase (see Fig 7B), on the other hand, new (previously unseen) examples are encoded into the thalamus through the same protocol of the training phase, yet no contextual signal is given to cortical neurons nor readout neurons. The novel, previously unseen thalamic pattern activates cortical neuron groups trained over similar training examples (small bit-distance), which in turn activate groups of readout neurons coding for the class over which they have been trained. The network classification answer can be inferred in two ways: one, unsupervised, as the class over which has been trained the subgroup of cortical neurons that is most actively firing; two, supervised, as the class associated with the most active subgroup of readout neurons.

Balanced mini-batch training The training method used in this work is based on the balanced-mini-batch scheme proposed by Shimizu et al. in [1]. The training set has been divided into mini-batches of 10 examples each. In addition, we compare the performance of our model using different training protocols among those proposed by Lomonaco et al. in [2]. As depicted in Fig A, we compare the network performances on the classification of examples randomly extracted from the whole dataset, considering three different protocols: 1) using balanced mini-batches, i.e. each of the digit classes is represented by one example in every batch; 2) each batch contains 10 examples of the same digit class, in order to evaluate the capability of the model to learn new classes incrementally; 3) every single training example in the batch is extracted randomly from the whole training set.

Sleep-like oscillatory dynamics The rhythmic activity displayed during the sleeping phase by the network is a property that emerges when adaptation is included in

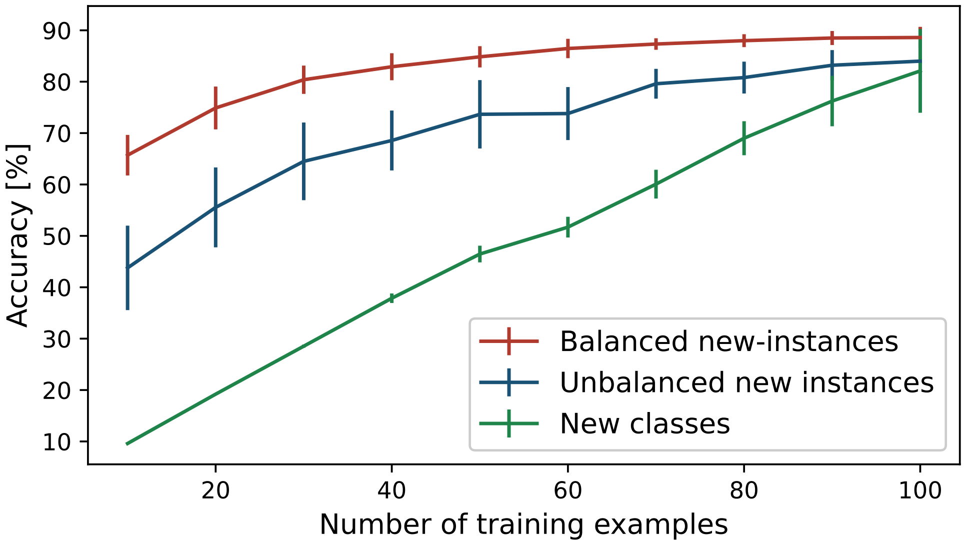

Fig A. Comparison of incremental learning protocols. The training set was divided into mini-batches of 10 examples each. Red line: mini-batches are balanced, i.e. each class is represented by one example in every batch. Blue line: every single training example in the mini-batch is extracted randomly from the whole training set. Green line: each batch contains examples of the same class, in order to evaluate the capability of the model to learn new classes incrementally. For all these methods, the accuracy of classification capability is evaluated over examples drowned from the whole testing dataset.

a model with two stable states characterized by low and high levels of activity (Down and Up respectively). If the parameters are chosen properly, the two states become metastable, and the system regularly oscillates between the two states. Sleep states are characterized by high levels of spike frequency adaptation currents. Under this condition, the firing rate in the network exhibits spontaneous oscillations between the two fixed points. Each neuron is represented by a dot in the top panel of Fig 3 in the main text. The progressive depletion in strength of recurrent synapses induced by depressive STDP produces a reduction in the current circulating in the network, and consequently in the adaptation value. This mechanistic chain is responsible for the decrease in SO frequency reported in panel B (top). Late sleep and after-sleep classification have low levels of input currents, due to sleep-mediated synaptic depression. Moreover, in order to verify that the homeostatic effect and the sharpening of the synaptic weight is a pivotal element for the beneficial effect of sleep, we handled the STDP asymmetry parameter α (see SI section, 'Spike-Timing-Dependent Plasticity' paragraph) and evaluated the impact on the network performances. Results of this analysis are depicted in Fig B: we train the network on 5 examples per category (50 examples total), and we measure the network performances before and after the deep-sleep-like phase (duration: 100s) with the thalamo-cortical connection cut out (thus no associative effect implemented). Specifically, in Fig BA, we show that for smaller values of the asymmetry parameter, the deep sleep activity leads to a drop in performances, while for values close to the ones used for this article (namely $\alpha \sim 5.$), it produces a beneficial effect on the network performances. Indeed, higher values of the asymmetry parameter determine a shorter total time for alternation of Up and Down states in Slow Oscillation cycles leading to reduced effects on the network. Moreover, we investigated how SO properties affect the post-sleep classification performances, shown in Fig BB. We did so by relating the magnitude of the STDP asymmetry parameter α on SO frequencies and durations, as shown in the inset. Also, we measured the homogenization effect on cortical firing rates distributions in classification phase and related it to the asymmetry parameter. In Fig BC we compare the network pre- and post-sleep classification perfromances with the firing rates homogenization effect and provide the relation between this and the asymmetry parameter in the inset.

Spike-Timing-Dependent Plasticity Within the framework of the Hebbian learning, the development of neural circuits based on correlated activity relies on two critical mechanisms: 1) activity-dependent synaptic modification and 2) competition between different synapses so that when some associated to a given postsynaptic neuron are strengthened, others are weakened. Experimental evidence from several different preparations suggests that both the sign and degree of synaptic modification arise from the repeated time-depending pairing of pre- and post-synaptic action potentials. Namely, presynaptic action potentials that follow postsynaptic spikes produce a long-term weakening of synapses. The largest changes in synaptic efficacy occur when the time difference between pre- and postsynaptic action potentials is small, with a sharp transition from strengthening to weakening as this time difference passes through zero. We call this form of synaptic modification spike-timing-dependent plasticity (STDP). Morrison et al. [3] and Sboev et al. [4] proposed a pair-based update law

$$\Delta w = \begin{cases} -W_- \cdot (\frac{w}{w_{\max}})^{\mu_-} \cdot \exp\left(-\frac{t_{\text{pre}}-t_{\text{post}}}{\tau_-}\right), & \text{if } t_{\text{pre}} - t_{\text{post}} > 0 \\ W_+ \cdot (1 - \frac{w}{w_{\max}})^{\mu_+} \cdot \exp\left(-\frac{t_{\text{pre}}-t_{\text{post}}}{\tau_+}\right), & \text{otherwise} \end{cases}$$

where $w_{\max}$ represents a limiting value for the weight and α in $W^- = \alpha W^+$ depicts the asymmetry parameter of depressing and increasing synaptic weights. The exponents μ_+ and μ_- vary in the range [0;1]. In the two extreme cases $\mu_{+/-} = 0$ and $\mu_{+/-} = 1$ the

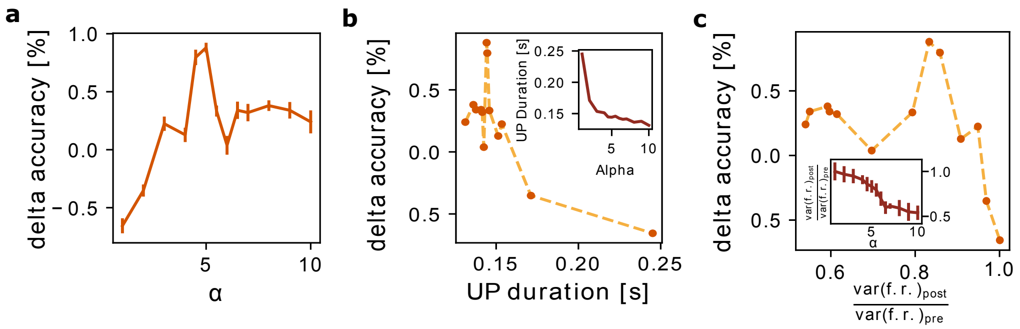

Fig B. Investigation of the deep-sleep effects on the network performances. **a**) Effect of 100s of deep-sleep-like activity on the classification performances as a function of the STDP-asymmetry parameter. Here, cortico-thalamic connections are removed to evaluate only the effect due to homeostasis and sharpening. For low values of α, the homeostatic effect is too strong, leading to a drop in performances. On the other hand, for high α no effects on network performances are measured. **b**) Impact of the duration of slow oscillations on network performances. Specifically, Up-states duration naturally decreases with alpha, as depicted in the inset. **c**) We evaluated the homogenization effect due to the sleeping phase as the ratio of post- and pre- sleep firing rate variance in the classification phase. As shown in the inset, this value is handled through the change of the asymmetry parameter α.

model is called additive STDP and multiplicative STDP, respectively. Throughout this work, we use a multiplicative STDP rule. According to the Hebb's postulate, since the weights of all thalamo-cortical synapses are plastic, if both the input pattern and the contextual signal are kept active for a sufficiently long time, the weights of synapses connecting active thalamic neurons to active cortical neurons will grow. Thus, the input pattern is learned throughout its encoding into thalamo-cortical synaptic weights. Likewise, synapses connecting the activated cortical excitatory neurons to the readout neurons coding for the right label are strengthened.

The neuron model The neuron model used in these simulations is a conductance-based adaptive-exponential integrate and fire model [5] implemented in NEST [6] (*aeif-cond-alpha neuron*). In this model, the time evolution of the membrane potential V is given by the first of the following differential equations. It incorporates a spike frequency adaptation mechanism, through the ω term. The time evolution of ω (second equation) captures the essential features of the neuronal fatigue that depends on the number of spikes emitted by the neuron itself in the recent past:

$$\begin{aligned} C_m \frac{dV}{dt} &= -g_L \left(V - E_L\right) + g_L \Delta_T e^{\frac{(V - V_{th})}{\Delta_T}} + I_{system} - \omega \\ \tau_\omega \frac{d\omega}{dt} &= a \left(V - E_L\right) + b \sum_k \delta(t - t_k) - \omega \end{aligned} \tag{1}$$

Moreover, whenever $V > V_{peak}$ the membrane potential is set to a reset value V_{reset}. Here, τ_ω is the adaptation time constant associated to the neuronal fatigue, C_m the membrane capacitance, E_l the reversal potential, V_T the threshold potential and Δ_T the exponential slope parameter, a term that has been introduced to make more accurate the modelling of the explosive rising toward the emission of the spike, once the threshold is surpassed. Moreover, a and b are the adaptation parameters. The current

in input to the neuron from excitatory and inhibitory neurons I_{system} can be written as:

$$I_{system} = g_{ex}(t)\,(E_{ex} - V) + g_{in}(t)\,(V - E_{in}) \tag{2}$$

where g_{ex} and g_{in} are the *time-dependent* excitatory and inhibitory synaptic conductance respectively shaped according to the *alpha-function*. The *alpha function* has been proposed [7] as a close match to the shape of postsynaptic potentials measured in vitro. It hence provides a biologically-plausible model for exploring the problem-solving abilities of spiking networks with temporal coding schemes. Assuming that one spike occurs at time t_s, the alpha function for the synaptic conductance is defined as

$$g(t) = \begin{cases} w\frac{(t-t_s)}{\tau_s}e^{-(t-t_s)/\tau_s} & \text{if } t > t_s \\ 0 & \text{if } t < t_s \end{cases} \tag{3}$$

This conductance has a gradual rise and a slow decay, peaking at $t_{max} = \tau_s$. According to this model, the instantaneous current injected by an incoming excitatory synapse spiking at $t_s = 0$ is

$$I(t) = w\frac{t}{\tau_s}e^{-t/\tau_s}(E_{ex} - V(t)) \tag{4}$$

The datasets of handwritten characters The MNIST database of handwritten digits consists of a training database of 60000 examples and a test database of 10000 examples. Digits have been size-normalized and centred in 28x28-pixels 8-bit grey-scale images [8]. Each digit is associated with a label, which is an integer between 0 and 9 that represents the correct output classification label, as defined by a human expert. The test subsets were randomly extracted from the MNIST test database, and they were used only for the final assessment of the accuracies. The number of examples in these subsets has been limited due to the high computation time of the simulations. In each experiment, we used a training subset containing 10 to 400 training examples. Since those numbers are relatively small, to ensure that each class is equally represented in the training samples, the training subsets were randomly extracted with a balanced-class representation constraint, i.e. 5 examples for each class in the 50-examples subsets and 20 examples for each class in the 200-examples subsets.

Thalamic coding of visual stimuli The Pre-processing algorithm is applied to the images to make them suitable to be encoded into the neural circuits. It is inspired by the computations that take place in the first layers of the visual system of the primates, but it is not a realistic description of them. First, the raw image is pre-processed through a deskewing algorithm; then, HOG filter is applied ($14x14$ cell size, $14x14$ block size, 9 orientation bins and $7x7$ block stride). We improved the protocol adopted in [9] by introducing a fuzzy-logic inspired approach that codes the HOG output into 6 truth values, each one stimulating a specific thalamic neuron, instead of the 4 values binning (see Fig C for a schematic description). The coding of each input feature from a continuous domain of possible intensities, e.g from the (0.0,1.0) range of possible values, into the discrete activity of subsets of individual thalamic neurons should preserve, at least partially, a distance-like notion (UltraLow: $[0; 0.25)$, MediumLow: $[0.25; 0.50)$, MediumHigh: $[0.50; 0.75)$, High: $[0.75, 1.0]$). Indeed, coding patterns of signals that are not too far in the original continuous domain should be more similar in their neuronal representations than patterns that represent more distant signals. Intuitively, preserving distances in the coding should induce benefits, in particular for networks trained with a small set of examples, both in the classification of novel images and in the retrieval of already seen ones. In retrieval, the second look perception of already seen examples would always be affected by noise. Each feature should be representative of a class of

neighbouring values, and this requires the representation of distances in the thalamic coding.

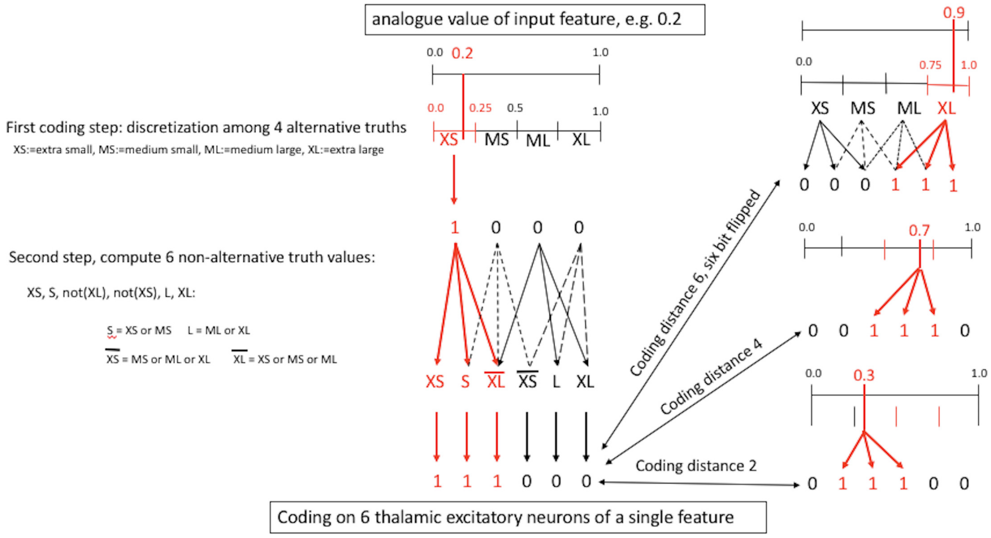

Fig C. Feature encoding. For each input feature, in this paper produced by the HOG filter in a continous domain, a binary encoding is implemented using 6 bit/feature. The adopted encoding introduces a distance-like metric, improving the 4 bit/feature encoding adopted in [9], but still keeping the simplifying assumption of having the same number of thalamic neurons firing for all values of the analog feature to be encoded. Proceeding top-down in the figure: an analog value (0.2) is encoded in a $\{1, 0, 0, 0\}$ pattern using 4 bits (**First coding step**). The Hamming distance is either 0 or 1 for all coding patterns. The **Second step** encodes the pattern in six bits, creating four possible Hamming distances $\{0, 2, 4, 6\}$ that correlate with the distance between original analogue values.

In our models, thalamic neurons are driven from the pre-processed visual stimuli to a *high* or *low* firing rate. Taking inspiration from the Fuzzy Logic [10] we defined a *distance* between two states of thalamic activity. Such distance is the number of thalamic neurons whose low or high firing rate state is required to be changed to move between two thalamic activity configurations. Fig C describes the proposed improvement. The novel 6-*values coding* uses the firing rates of 6 thalamic neurons to encode the value of each HOG feature. According to the 6-values coding, the distance between two possible states can be equal to $1, 2, 3$ per coded HOG feature instead of 1 as in [9]. This creates a smoother representation of the HOG information and reduces the loss we make whenever we discretize real values. The binned feature array is now ready to be given as input to the neuronal network: each Poisson generator connected to the input neurons is either activated with a fixed rate or switched off, depending on the corresponding bin value, 1 or 0 respectively.

Salt-and-pepper noise *Salt and pepper* noise refers to a wide variety of processes that result in the same basic image degradation: the number of noisy pixels is low but noise magnitude is quite high. The effect is similar to sprinkling white and black dots – salt and pepper – on the image. *Salt and pepper* noise is a good representation of the noise introduced when transmitting images over noisy digital links. In our scenario, the 2% of the pixels have been set to black or white with equal probability, whereas the others remain unchanged.

Parameters of the spiking model The network parameter optimization was done using a very small set of only four examples, due to the high number of such parameters and to the very long calculation times (a training procedure on a single subset of 200 examples followed by a validation on 500 examples, performed on the execution platform, took about 12.6 hours). For this optimization, a tuning tool was used that binds the rates of active neurons to stay within pre-established intervals, chosen to be compatible with the rate of excitatory and inhibitory neurons of the cortex, and minimizes the rate of the $N_{\text{exc}} - k$ neurons not belonging to group D(l) of the k neurons that produce the highest signal. In order to achieve the equilibrium distribution of the plastic connection weights, the product between the learning rate and the time course of the simulated neuronal activity must be sufficiently large. However, if the learning rate is too high, the weight distributions will be affected by significant fluctuations. By performing multiple simulations on the same training / validation subsets, with fixed parameters, varying only the seed for the generation of pseudo-random numbers, we observed that using a 40-second neuronal activity time for each training example the fluctuations in accuracy were less than 2%. The network parameters used in the simulations are shown in Table A.

References

1. Shimizu R, Asako K, Ojima H, Morinaga S, Hamada M, Kuroda T. Balanced Mini-Batch Training for Imbalanced Image Data Classification with Neural Network. In: 2018 First International Conference on Artificial Intelligence for Industries (AI4I); 2018. p. 27–30.

2. Lomonaco V, Maltoni D. CORe50: a New Dataset and Benchmark for Continuous Object Recognition. In: Levine S, Vanhoucke V, Goldberg K, editors. Proceedings of the 1st Annual Conference on Robot Learning. vol. 78 of Proceedings of Machine Learning Research. PMLR; 2017. p. 17–26. Available from: http://proceedings.mlr.press/v78/lomonaco17a.html.

3. Morrison A, Diesmann M, Gerstner W. Phenomenological models of synaptic plasticity based on spike timing. Biological Cybernetics. 2008 Jun;98(6):459–478. Available from: https://doi.org/10.1007/s00422-008-0233-1.

4. Sboev A, Vlasov D, Serenko A, Rybka R, Moloshnikov I. On the applicability of STDP-based learning mechanisms to spiking neuron network models. AIP Advances. 2016;6(11):111305. Available from: https://doi.org/10.1063/1.4967353.

5. Brette R, Gerstner W. Adaptive Exponential Integrate-and-Fire Model as an Effective Description of Neuronal Activity. Journal of Neurophysiology. 2005;94(5):3637–3642. PMID: 16014787. Available from: https://doi.org/10.1152/jn.00686.2005.

6. Kunkel S, Morrison A, Weidel P, Eppler JM, Sinha A, Schenck W, et al.. NEST 2.12.0. Zenodo; 2017. Available from: https://doi.org/10.5281/zenodo.259534.

7. Rall W. Distinguishing theoretical synaptic potentials computed for different soma-dendritic distributions of synaptic input. Journal of Neurophysiology. 1967;30(5):1138–1168. PMID: 6055351. Available from: https://doi.org/10.1152/jn.1967.30.5.1138.

Number of neurons	k_{cx}<br>n_{cx}<br>n_{inh}<br>n_{th}	20<br>$k_{cx} \cdot N\_Classes \cdot N\_Ranks$<br>$n_{cx}/4$<br>486	k_{ro}<br>n_{ro}<br>n_{re}	20<br>$k_{ro} \cdot N\_Classes$<br>200
Static synapses weights	$W_{\mathrm{th}\to\mathrm{re}}$<br>$W_{\mathrm{cx}\to\mathrm{inh}}$	3.0<br>200	$W_{\mathrm{re}\to\mathrm{th}}$<br>$W_{\mathrm{inh}\to\mathrm{cx}}$	-1<br>-1
Plastic synapses initial weights	$W_{0;\mathrm{th}\to\mathrm{cx}}$<br>$W_{0;\mathrm{cx}\to\mathrm{cx}}$<br>$W_{0;\mathrm{ro}\to\mathrm{ro}}$	0.3<br>0.01<br>0.01	$W_{0;\mathrm{cx}\to\mathrm{th}}$<br>$W_{0;\mathrm{cx}\to\mathrm{ro}}$	0.1<br>0.01
Plastic synapses maximum weights	$W_{\max;\mathrm{th}\to\mathrm{cx}}$<br>$W_{\max;\mathrm{cx}\to\mathrm{cx}}$<br>$W_{\max;\mathrm{ro}\to\mathrm{ro}}$	10.5<br>20.0<br>20.0	$W_{\max;\mathrm{cx}\to\mathrm{th}}$<br>$W_{\max;\mathrm{cx}\to\mathrm{ro}}$	0.333<br>675
Awake neuron parameters	t_{train} (ms)<br>α<br>τ_ω	1500.0<br>1.0<br>144.0	t_{test} (ms)<br>b	1000.0<br>0.01
Plastic synapses awake learning rate	$\lambda_{\mathrm{th}\to\mathrm{cx}}$<br>$\lambda_{\mathrm{cx}\to\mathrm{cx}}$<br>$\lambda_{\mathrm{ro}\to\mathrm{ro}}$	0.03<br>0.1<br>0.1	$\lambda_{\mathrm{cx}\to\mathrm{th}}$<br>$\lambda_{\mathrm{cx}\to\mathrm{ro}}$	0.003<br>0.03
Poisson noise awake weights	$W_{\mathrm{noise}\to\mathrm{th}}$<br>$W_{\mathrm{noise}\to\mathrm{inh}}$	1600<br>320	$W_{\mathrm{noise}\to\mathrm{cx}}$<br>$W_{\mathrm{noise}\to\mathrm{ro}}$	1100<br>1100
Poisson noise awake rates	$r_{\mathrm{noise}\to\mathrm{th}}(\mathrm{s}^{-1})$<br>$r_{\mathrm{noise}\to\mathrm{inh}}(\mathrm{s}^{-1})$	40.0<br>80.0	$r_{\mathrm{noise}\to\mathrm{cx}}(\mathrm{s}^{-1})$<br>$r_{\mathrm{noise}\to\mathrm{ro}}(\mathrm{s}^{-1})$	80.0<br>80.0
Sleep parameters	t_{sleep} (s)<br>$W_{\mathrm{noise}\to\mathrm{cx}}$<br>$\tau_w$	200.0<br>18.0<br>400.0	$r_{\mathrm{noise}\to\mathrm{cx}}(\mathrm{s}^{-1})$<br>$b$<br>$\alpha$	650.0<br>150.<br>5.0
Plastic synapses sleep learning rate	$\lambda_{\mathrm{th}\to\mathrm{cx}}$<br>$\lambda_{\mathrm{cx}\to\mathrm{ro}}$	0.0<br>0.0	$\lambda_{\mathrm{cx}\to\mathrm{th}}$<br>$\lambda_{\mathrm{cx}\to\mathrm{cx}}$	0.0<br>0.0008
Other Parameters both awake and sleep	C_m (pF)<br>$E_L(mV)$<br>$V_{th}(mV)$<br>$a(ns)$	281.0<br>-70.6<br>-50.4<br>4.0	$g_L(nS)$<br>$\Delta_T(mV)$<br>$V_{peak}(mV)$	30.0<br>2.0<br>0.0

Table A. Parameters of the simulations. $N_{Classes}$ is the number of digits classes (typical case: 10 classes) over which the network has been trained; N_{Ranks} is the number of incremental learning cycles (in this work, from 1 to 20); k depicts the number of neurons selected by the contextual signal in the training phase. The subscript *th* refer to the excitatory population of the thalamic input layer; *cx* refer to the excitatory population of the cortical layer; *ro* to the readout layer; *inh* and *re* refer to the inhibitory populations of the thalamic and cortical layer, respectively; *noise* to the Poisson signals that are sent to different populations; r represents the rate of those signals; W represents the synaptic weight; W_0 is the initial weight of STDP synapses; W_{max} is the maximum weight of STDP synapses; λ is the learning rate of STDP synapses. Poissonian noises are all set at zero during the sleeping phase with the only exception of the one provided to *cx* neurons.

8. LeCun Y, Cortes C, Burges CJC. The MNIST database of handwritten digits;. "http://yann.lecun.com/exdb/mnist/".

9. Capone C, Pastorelli E, Golosio B, Paolucci PS. Sleep-like slow oscillations improve visual classification through synaptic homeostasis and memory association in a thalamo-cortical model. Scientific Reports. 2019 June;9:8990. Available from: https://https://www.nature.com/articles/s41598-019-45525-0.

10. Zadeh LA. Fuzzy logic. Scholarpedia. 2008;3(3):1766. Revision #123810.
