# Supplementary material for: Thalamo-cortical spiking model of incremental learning combining perception, context and NREM-sleep
Source: PLoS Comput Biol. 2021 Jun 28;17(6):e1009045. doi: 10.1371/journal.pcbi.1009045 (PMC8270441; doi:10.1371/journal.pcbi.1009045)
Supplement: Attachment — Submitted filename: ResponseReviewers.pdf [file pcbi.1009045.s002.pdf]

Dear Editor,

We re-submit the article “Thalamo-cortical spiking model of incremental learning combining perception, context and NREM-sleep-mediated noise-resilience” following the accurate and valuable revision carried out by your journal.

We are grateful to the reviewers for their questions, specific suggestions for improvements and requests for the addition of material on topics that, indeed, were not sufficiently explained in the original submission; in our opinion, the quality of the manuscript greatly improved thanks to the inputs provided by the reviewers and the new elements stimulated by their productive indications. We also modified the table of parameters (Table 2) to make it more legible. In the following, we divided the questions raised by the reviewers into points (named RxQy): we first provide a synthetic answer to each question, followed by the text that we purpose for addition to the manuscript. We kindly ask the Editor to share this letter with both the reviewers, since some of the answers can be addressed to both of them and are cross-referenced in the replies. In the manuscript, the text proposed for addition is marked in red. We have created and currently populating a repository in GitHub (pointed at line 525 in the manuscript) to make all data used to draw the conclusions outlined in this paper available.

Responses to reviewer's questions

Reviewer #1: *In this study, the authors use a spiking model of thalamocortical network that is capable of learning to examine the role of sleep-like state. Using the oscillatory input that induced activity in the thalamocortical network for sleep state, the study finds that it improves subsequent learning. Overall, the study identifies interesting results, however requires several issues to be addressed.*

- **R1Q1**: *The description of the sleep state is not presented in the manuscript. The author's refer to their previous publication in the main text and include very few details about sleep in the methods section. Please include additional details about how sleep is implemented in both main text and also in the methods.*

We propose the addition of specific text, distributed among the <Introduction> and <Results> sections (lines 91, 203 respectively), complemented by three panels in Fig 3 that illustrate the main characteristics of the sleep induction methods and effects. With this additional text, we provide more details about the method used to induce the model into deep-sleep, and about the resulting emergent rhythmic activity with consequent spontaneous homeostatic and associative effects, summarizing the main results of our previous work by Capone et al. 2019.

For the sake of convenience, we report here the text added to the paper and the new Figure provided:

Line 91

Indeed, in [Capone et al. 2019], some of us illustrated how to assemble a simplified thalamo-cortical spiking model that can both express deep-sleep-like oscillations (in the form of an emergent, self-induced network phenomenon) and enter an awake-like asynchronous regime. This dynamical behaviour has been obtained by changing a few parameters in the

equation that describes the dynamics of excitatory neurons in the spiking model and thus exploiting a well established modelling principle that represents a few prominent features of brain-state acetylcholine-mediated neuromodulation, able to induce in the model the transition between awake-like asynchronous and deep-sleep-like oscillatory regimes [Goldman et al. 2019]. However, this neuromodulation modelling principle has neither been previously applied to the study of the deep-sleep cognitive effects nor to simulations of learning-sleep cycles (such as in our previous work [Capone et al. 2019] and in this study). Specifically, the spiking model we propose is trained on a set of training patterns (here, on images of handwritten digits) and then exposed to never-seen examples to be classified (here, among human-assigned digit classes). Also, the model structure proposed in [Capone et al. 2019] and adopted in this work, is able to perform an asynchronous awake-like state of the network, by acting on the neural dynamics parameters. When the prescribed changes in the neural parameters induce the network to express deep-sleep-like oscillations, STDP is observed to produce in the model the spontaneous emergence of a differential homeostatic process. First, a down-regulation emerges of the stronger synapses created by the STDP during the training, those synapses that connect the best-tuned neurons during the training phase on each training example. At the same time, we observe that STDP increases the strength of synapses among neurons tuned on patterns belonging to the same class. Such hierarchical, spontaneous reorganization promotes better post-sleep classification performances. In short, the underlying mechanism is based on the similarity among thalamic coding of training examples belonging to the same class. During deep-sleep oscillations, such similarity supports the preferential activation of thalamo-cortico-thalamic connection paths among neural groups tuned to training examples belonging to the same class, and the consequent coactivation and unsupervised strengthening of class-specific synapses. This point has been illustrated in Fig. 5 Panel A of [Capone et al. 2019].

Line 203

When approaching the design of the ThaCo spiking model, an improvement of what some of us presented in [Capone et al. 2019], we relied on the well-established framework of Mean-Field theories [Gigante et al 2017, Capone et al 2017], to construct a network capable of spontaneously displaying two different dynamical regimes. This is obtained by acting on some parameters of the excitatory neurons (specifically, the spike frequency adaptation (SFA) and the excitatory synaptic conductance), to model acetylcholine-mediated neuromodulation on neural dynamics that supports the transitions between awake-like asynchronous activity and deep-sleep-like slow oscillations [Goldman et al 2019]. Specifically, in Fig.3A we show the incoming current to each cortical neuron versus its adaptation current during different network stages (pre- and post-sleep classification, beginning and end of the sleeping phase). In particular, sleep states are characterized by high levels of spike frequency adaptation currents (obtained through a modulation of the SFA parameter), inducing oscillations. Moreover, late sleep and after-sleep classification have low levels of input currents, due to a sleep-mediated synaptic depression leading to a reduction in the current circulating in the network. When set in the deep-sleep state, a non-specific stimulus, administered at a low steady firing rate to cortical neurons, is sufficient to elicit the emergence of cortically-generated Up-states and of thalamo-cortical Slow Oscillations (SO). As shown in top lines of Fig.3B and C, in the SO regime, the thalamo-cortical spiking network displays a firing rate oscillation frequency between 0.25 Hz and 1.0 Hz and

durations of Up-states (a few hundreds of ms) comparable with experimental observations in deep-sleep recordings. During the initial stages of SO, Up-states are independently sustained by neuron populations tuned to specific images memorized during the training phase, and tend to reactivate thalamic neuron coding for the memorized images. Then, thanks to the similarity among training instances, the recruitment of other neural groups in the cortex is promoted. This creates preferential cortico-talamo-cortical excitatory pathways, inducing an STDP-mediated association of cortical neurons previously tuned to training instances that expressed similar thalamic representation (see Fig. 3B and C). We name *top-down prediction* such cortico-thalamic activation that spontaneously occurs during SO. During the sleep period, thanks to cortico-cortical plasticity, the coactivation of neurons originally tuned to training instances of the same class becomes a typical feature of each Up-state: the WTA mechanism cooperates in selecting different neuron codings for different classes during each Up-State. Another key aspect is the generalized homeostatic depression, which is known to happen during deep-sleep and serves as a protection, to prevent Up-state-mediated associations that could drive towards a fully associated network. This effect is modelled thanks to the Non-Linear Temporal Asymmetric Hebbian (NLTAH) learning rule of the STDP we used [Gütig et al 2003], which reduces the strength among the most frequently coactivated neurons, leading to a progressive reduction of mean firing rates and frequency of the Up-States (see Fig. 3B and C, top rows) which is consistent with experimental observations, in particular for what concerns the decrease of SO frequency during the night course [Hobson et al. 2002]. The first noteworthy result presented in this paper is that this new calibration of the model greatly enhances the match with experimental data, as detailed in the following. Indeed, while the model [Capone et al. 2019] was already able to express the transition between states [Tort-Colet et al 2019] such as sleep-like slow oscillations activity and awake-like classification, the refinements of its parameters here introduced make ThaCo more biologically plausible, leveraging as calibration tool the accurate comparison with experimental observations of differential changes in firing rates.

Figure 3 - new version

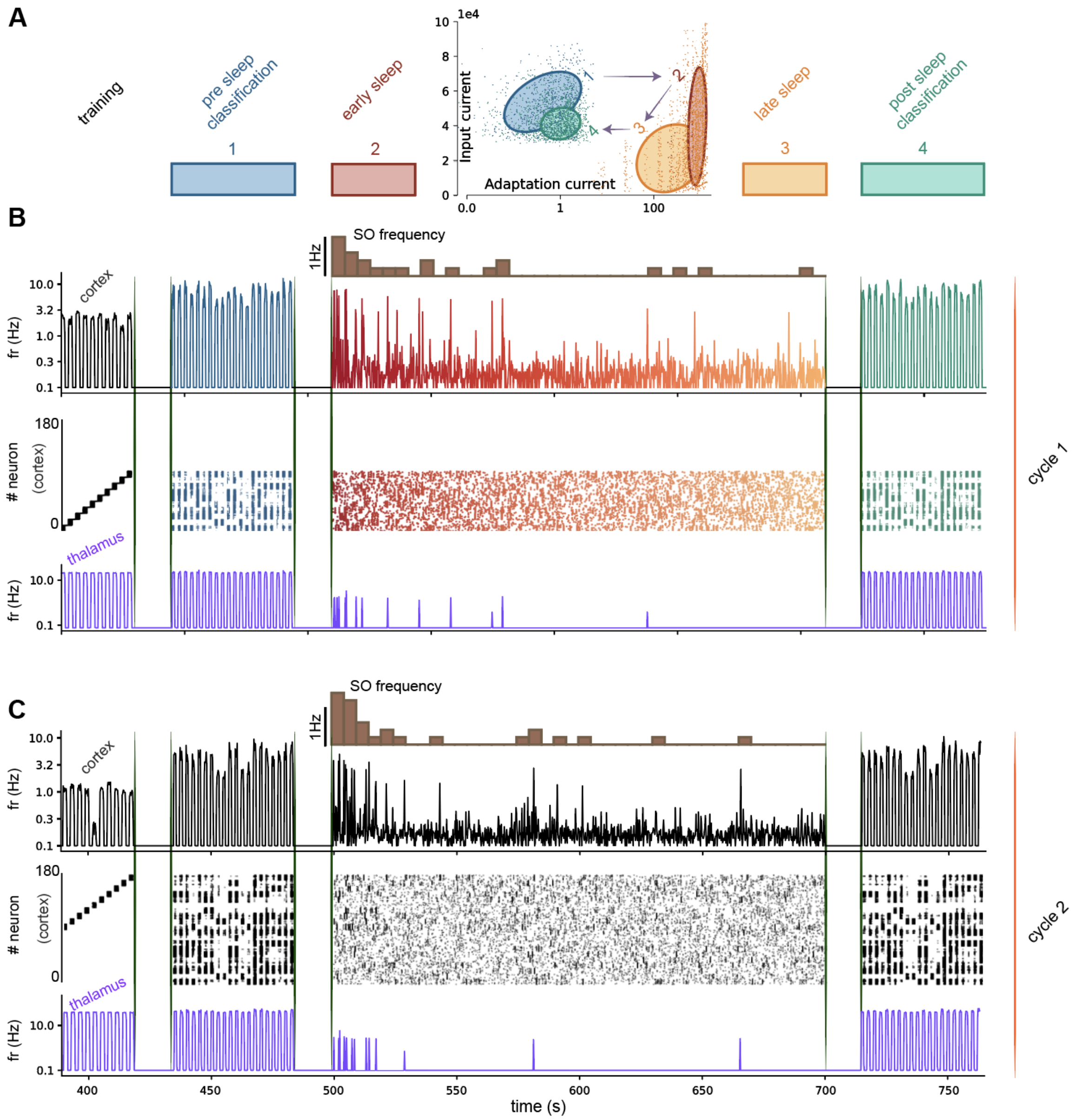

Figure 3. Incremental learning with alternation of awake training and sleep in ThaCo. **A)** *Dots:* incoming current to each cortical neuron versus its adaptation current during different network stages of the activity presented in B. Pre-sleep classification phase (represented in blue, number 1), early sleep-like phase (in red, number 2), late sleep-like phase (in orange, number 3) and post-sleep classification phase (in green, number 4). *Ellipses*: Areas in the plot associated with different network stages, estimated from data through a Gaussian Mixture Model with a full covariance matrix. During sleep, the total input current to the cortical neurons decreases due to the sleep-induced homeostatic effect, that reduces recurrent connections weights in the cortical layer (see the transition from number 2 to number 3 in the diagram), notwithstanding the constant external aspecific stimulus. Sleep-like activity, on the other hand, affects the network status during the following awake classification phase 4: the effect of STDP during sleep is a general reduction and homogenization of input current distribution, as shown in a comparison between the pre-sleep stage 1 and post-sleep stage 4 in the diagram. **B** and **C**) Spiking cortical and thalamic activity produced during training (10 examples, one per digit class), classification

(20 images) and sleeping phase for two consecutive sets respectively. First row: mean firing rate of the cortical neurons trained over a set of 10 examples (each set is used to independently train 200 cortical neurons, 20 per digit example); during the sleeping phase, the slow oscillation frequency trend in time is also depicted. Second row: raster plot of the first 400 cortical neurons. Third row: mean firing rates of thalamic neurons. Once recruited in the training phases, the cortical neurons participate in classification and sleeping phases.

- ***R1Q2**: Based on author's previous paper, I assume sleep involves oscillatory input applied to cortical neurons. How does the frequency and duration of the oscillation impact the results?*

We remark that SOs are not induced by an external oscillatory input but are an endogenous phenomenon expressed by the simulated network. This is explained by the presence of a limit cycle in the population dynamics [Gigante et al 2017, Capone et al 2017]. To better explain this, we have added a new section in the supporting information section (“Sleep-like oscillatory dynamics” line 684) aiming at illustrating the setup and implementation of the sleeping dynamics. We also investigated how SO properties affect the result by changing the STDP asymmetry parameter α (as depicted in the new Figure 9 in Supporting Information), which induces different SO frequencies and durations. This result is specifically summarised in panel B of this figure, also reported here.

Supporting information section - “Sleep-like oscillatory dynamics”, line 709

Moreover, we investigated how SO properties affect the post-sleep classification performances, shown in Fig.9B. We did so by relating the magnitude of the STDP asymmetry parameter α on SO frequencies and durations, as shown in the inset.

Figure 9 - panel B:

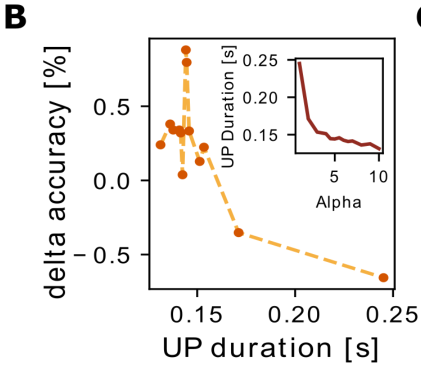

Effects of deep-sleep activity on network performances investigation. […] **B**) Impact of the duration of slow oscillations on network performances. Specifically, Up-states duration naturally decreases with alpha, as depicted in the inset.

- **R1Q3**: *Another critical issue in the manuscript is about what is referred as incremental learning. In this manuscript, incremental learning refers to increasing the number of training sets across all classes. However, typically the term incremental learning (often used in machine learning) involving tasks such as MNIST involve learning different classes incrementally. While this distinction may be subtle, it is critical since learning classes is known to lead to forgetting.*

We have expanded the text with a brief description of how different approaches to incremental learning and different learning schemes are classified in the context of machine learning. To better frame our approach, see lines 130 and 162. We made some new simulations in which we evaluated the performance of the proposed models on different learning schemes, including learning new classes incrementally. We describe these results by introducing a new paragraph in the Supporting Information Section named “Balanced mini-batch training” (line 673) and adding a new figure (Fig. 8). However, we also emphasize that our model is not intended as an engineering solution to the problem of incremental learning, but as a simplified model of the low-level processes that support this capability in the biological brain, and that many issues that are of primary importance for both biological and artificial incremental learning go beyond the aims of this work, see lines 348.

For the sake of convenience, we report here the add text to the paper and the new Figure provided:

Line 130:

In the context of machine learning, a distinction is made between instance-incremental methods, which learn from each training example as it arrives, and batch-incremental methods, in which the training data are organized in groups of examples, called batches, and the model is trained only on complete batches [Read et al 2012]. Depending on how different classes are represented by the examples in the batches, there are three training schemes [Lomonaco and Maltoni 2017]: new instances (NI), in which each new batch contains different instances of the same classes represented in previous batches, new classes (NC) in which examples belonging to novel classes become available in subsequent batches, and new instances and classes (NIC) in which subsequent training batches contain examples from both known and new classes. However, it should be noted that when it is necessary to constantly evaluate the performance of incremental learning, for reasons of computational efficiency the training set is divided into batches even for models capable of instance-incremental learning. Shimizu et al. [Shimizu et al. 2018] propose a training method based on balanced mini-batches, which reduces the effect of imbalanced data in supervised training. Our work is focused on instance incremental learning, and the training scheme is based on balanced mini-batches.

Line 162:

After the validation obtained against experimental results, we demonstrate the capability of the model to learn incrementally, i.e. to continuously extend its knowledge by learning from new training examples while retaining most of the previously acquired memories. [...] It should be emphasized that the proposed model is not an engineering solution to the problem of incremental learning in pattern classification, but a simplified model of the low-level processes that supports and emulates the ability to learn incrementally in the biological brain. Indeed, the size of the training set is relatively small compared to those often used in machine learning, and some issues that are of primary importance for both artificial and biological incremental learning, such as catastrophic forgetting, go beyond the aims of this paper.

Line 348:

The model presented in this work enables instance-incremental as well as class-incremental learning. The training protocol we adopted for the results presented here was based on the balanced-mini-batches scheme proposed by [Shimizu et al. 2018]. More specifically, the training set of hand-written digits was divided into mini-batches of 10 examples each, in which each class was represented by just one example. In Fig. 8 in Supporting Information, we include a comparison of performance obtained using different training protocols.

Supplementary Information - “Balanced mini-batch training”, Line 673:

The training method used in this work is based on the balanced-mini-batch scheme proposed by Shimizu et al. in [Shimizu et al. 2018]. The training set has been divided into mini-batches of 10 examples each. In addition, we compare the performance of our model using different training protocols among those proposed by Lomonaco et al. in [Lomonaco and Maltoni 2017]. As depicted in Fig.8, we compare the network performances on the classification of examples randomly extracted from the whole dataset, considering three different protocols: 1) using balanced mini-batches, i.e. each of the digit classes is represented by one example in every batch; 2) each batch contains 10 examples of the same digit class, in order to evaluate the capability of the model to learn new classes incrementally; 3) every single training example in the batch is extracted randomly from the whole training set.

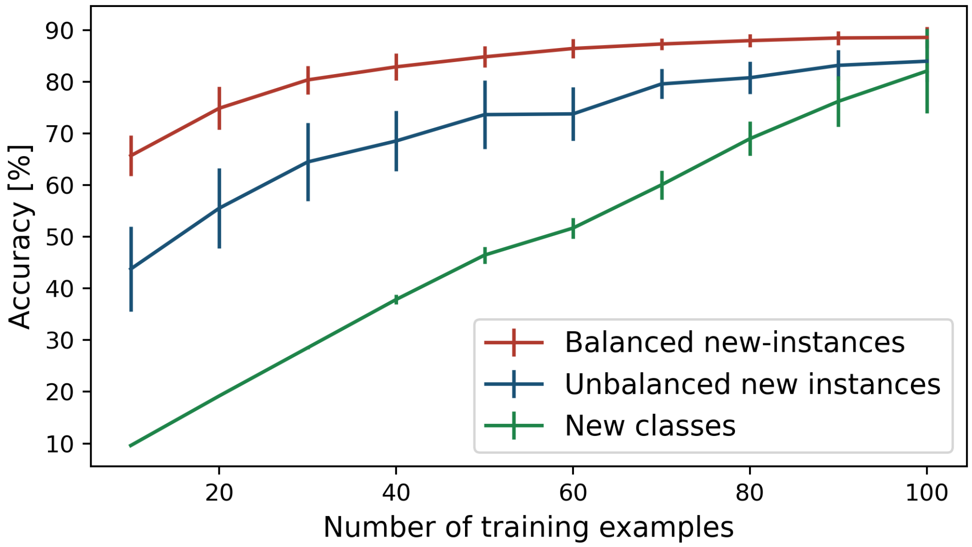

Comparison of incremental learning protocols. The training set was divided into mini-batches of 10 examples each. Red line: mini-batches are balanced, i.e. each class is represented by one example in every batch. Blue line: every single training example in the mini-batch is extracted randomly from the whole training set. Green line: each batch contains examples of the same class, in order to evaluate the capability of the model to learn new classes incrementally. For all these methods, the accuracy of classification capability is evaluated over examples drowned from the whole testing dataset.

- **R1Q4**: *The authors suggest that homeostatic effect and sharpening of the synaptic weight is critical for the beneficial effect of sleep. It would be helpful to try manipulation of the homeostatic effect and sharpening by changing the STDP rules, and examining the results. This way, the postulated mechanisms could be verified.*

We have expanded the text (line 431) with a reference to a new section in the Supporting information section (paragraph "Sleep-like oscillatory dynamics" line 684) aiming at illustrating that the combination of homeostatic effect and sharpening of synaptic weights leads to an improvement in classification performance. To do so, we made some new simulations in which we modified the asymmetry parameter and neglected the associative effect of sleep. We show that for smaller values of the asymmetry parameter, deep sleep activity leads to a drop in performances, while for values close to the ones used for this article the homeostasis produces a beneficial effect on the network performances; higher values of the asymmetry parameter determine a shorter total time for the alternation of Up and Down states in Slow Oscillation cycles leading to reduced effects on the network. These results are depicted in the new Figure 9 in the Supplementary Information section. We also modified line 730 in the Supporting information section providing a more convenient definition of the asymmetry parameter in the STDP rule.

For the sake of convenience, we report here the text added to the paper and the new Figure provided:

Line 431

Asymmetric STDP induces, on one hand, the depression of strong synapses, on the other the association among neuronal groups coding for the same class (i.e. trained over similar stimuli), through a mechanism of resemblance in their thalamic representation.

Supporting information section - "Sleep-like oscillatory dynamics", line 697

In order to verify that the homeostatic effect and the sharpening of the synaptic weight is a pivotal element for the beneficial effect of sleep, we handled the STDP asymmetry parameter α (see SI section, paragraph "Spike-Timing-Dependent Plasticity") and evaluated the impact on the network performances. Results of this analysis are depicted in Fig9: we train the network on 5 examples per category (50 examples total), and we measure the network performances before and after the deep-sleep-like phase (duration: 100s) with the thalamo-cortical connection cut out (thus no associative effect implemented). Specifically, in Fig.9A, we show that for smaller values of the asymmetry parameter, the deep sleep activity leads to a drop in performances, while for values close to the ones used for this article (namely α~5.0), it produces a beneficial effect on the network performances. Indeed, higher values of the asymmetry parameter determine a shorter total time for the alternation of Up and Down states in Slow Oscillation cycles leading to reduced effects on the network.

Figure 9 - panel A:

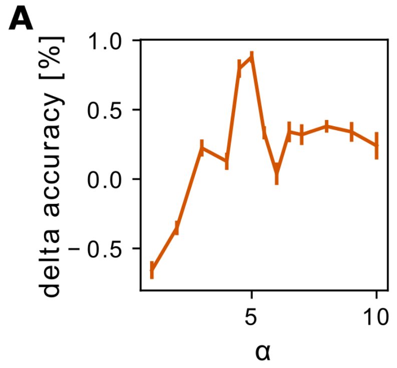

Effects of deep-sleep activity on network performances investigation. **A**) Effect of 100s of deep-sleep-like activity on the classification performances as a function of the STDP-asymmetry parameter. Here, cortico-thalamic connections are removed to evaluate only the effect due to homeostasis and sharpening. For low values of α, the homeostatic effect is too strong, leading to a drop in performances. On the other hand, for high α no effects on network performances are measured.

- **R1Q5**: *It is not clear what is the role of inhibitory neurons and TC and RE cells in thalamus during sleep?.*

In our model, the activation of cortico-thalamo-cortical pathways is essential for the association of memories belonging to the same class. This effect is due to the higher similarity among such memories, with inhibition playing a key role in supporting the selection of active neural groups. This is demonstrated in Fig. 5 panel A of [Capone et al.2019]. We clarified this point with the addition of text already proposed to answer Q1R1, reported here:

Line 116

During deep-sleep oscillations, such similarity supports the preferential activation of thalamo-cortico-thalamic connection paths among neural groups tuned to training examples belonging to the same class, and the consequent coactivation and unsupervised strengthening of class-specific synapses. This point has been illustrated in Fig. 5 Panel A of [Capone et al. 2019].

- **R1Q6**: *Biological experiments, suggest that inhibitory and thalamic neurons play important role in synchronization across regions, and is helpful to examine their role in this network.*

We thank the reviewer for raising this very interesting point. However, the modelling of different brain regions is out of the scope of this work also due to 1) the extremely small size of the explored network, and 2) the consequently short synaptic delays used in the simulation. We specified this in the discussion section as one of its limits, as follows:

Line 497 - Discussion

One more limitation of this work is that it does not take into account the role of synchronization among different brain regions. Actually, assuming a typical neural density in

the range of $5x10^4$ neurons per mm^2 of the cortex, and considering that the maximum size of the proposed model rises up to 5000 cortical neurons, such a number is equivalent to a small cortical area with a dimension of about 300μm, that is well below the size of a single cortical area. To overcome this limitation

----

We thank the reviewers for their questions, specific suggestions for improvement and requests for the addition of material on specific topics that, indeed, were not sufficiently explained in the original submission. In our opinion, the quality of the manuscript greatly improved thanks to the addition of the material added to clarify the point raised by the reviewers. In the following, we divided the questions raised by the reviewers into points (named RxQy). Then, we first provide a synthetic answer to each question, followed by the text that we purpose for addition in the manuscript. In the manuscript, the text proposed for addition is marked in red.

Reviewer #2: *I applaud the efforts of the authors to create a model inclusive of cortical structure, thalamocortical interactions, STDP, sleep homeostasis and other elements. The paper appears to have some interesting observations and appears to be based on a great deal of work modifying and old model. However, many crucial details appear not to be explained, rendering it impossible for me, as a biologist, to understand the basic experiments done here. Those need to be clarified before I can fully evaluate this paper.*

MAJOR ISSUES:

- **R2Q1**: *How was the spike rate homogenization shown in Figure 2 instantiated? Why did this occur? Did it naturally fall out of the network with no intentional design? Was it explicitly added to re-create the biological situation? I find a great deal is lacking in the description of how this was carried out.*

The spike rate homogenization emerges from the combination of the spontaneous dynamics of the model with the adopted model of plasticity. Relying on experimental observations [Gonzales et al. 2018, Tononi 2019] we used an asymmetric form of plasticity during deep sleep dynamics, namely the NLTAH (Non-Linear Temporal Asymmetric Hebbian) form of STDP (see [Gütig, et al 2003]). This ingredient was enough to induce homogenization of synapses and activity homogenization. During sleep, indeed, asymmetric STDP induces the depression of the strongest synaptic weights, leading to a progressive reduction of mean firing rates and frequency of the Up States (see Figure 3, panel A) which is consistent with the experimental observations we compare to [Watson et al 2016]. Notwithstanding the depression of strong synapses, the network dynamics spontaneously creates also an association among neuronal groups coding for the same class (i.e. trained over similar stimuli), through a mechanism or resemblance in their thalamic representation (described at line 431). This is the mechanism behind the homogenization of firing rates and synaptic weights, coherently with [Watson et al 2016]. We claim that this feature naturally emerges from a biologically-plausible mechanism that we include in the simulation.

To better explain the homeostatic effect of sleep and verify its role in improving the network performance, we have expanded the text with a reference to a new section in the Supporting information section (“Sleep-like oscillatory dynamics”, line 684).

Please, see also our answer to the related question R1Q1 posed by the other reviewer.

Line 431

Asymmetric STDP induces, on one hand, the depression of strong synapses, on the other the association among neuronal groups coding for the same class (i.e. trained over similar stimuli), through a mechanism of resemblance in their thalamic representation.

Supporting information section - “Sleep-like oscillatory dynamics”, line 697

In order to verify that the homeostatic effect and the sharpening of the synaptic weight is a pivotal element for the beneficial effect of sleep, we handled the STDP asymmetry parameter α (see SI section, paragraph “Spike-Timing-Dependent Plasticity”) and evaluated the impact on the network performances. Results of this analysis are depicted in Fig9: we train the network on 5 examples per category (50 examples total), and we measure the network performances before and after the deep-sleep-like phase (duration: 100s) with the thalamo-cortical connection cut out (thus no associative effect implemented). Specifically, in Fig.9A, we show that for smaller values of the asymmetry parameter, the deep sleep activity leads to a drop in performances, while for values close to the ones used for this article (namely α~5.0), it produces a beneficial effect on the network performances. Indeed, higher values of the asymmetry parameter determine a shorter total time for the alternation of Up and Down states in Slow Oscillation cycles leading to reduced effects on the network.

- **R2Q2**: *It would be logical to test whether the log-normal distribution of neuronal firing rates was crucial to the network results here. That appears to have been specifically added here, but it was not shown whether it was important*

We note that lognormal distributions emerge from our simulations, they have not been pre-imposed. To better explain this, we added the following text in the paper:

Line 263

An interesting feature is that lognormal distributions spontaneously emerge from our simulations. This result is coherent with experimental observations and with theoretical considerations showing that the lognormal distribution of activities in randomly connected recurrent networks is a natural consequence of the non-linearity of the input-output gain function [Roxin et al. 2011].

- **R2Q3**: *Similarly, the effect of homogenization during sleep was added specially here, but is not investigated*

As discussed in the previous answer R2Q1, homogenization is induced by asymmetric STDP through the modulation of the collective α parameter (a proxy for the modelling of acetylcholine neuromodulation recently adopted by modellers [Gütig et al 2003]). In the manuscript, we observe that homogenization has a crucial role in the improvement of after-sleep performances (see Section 2.5, Fig 5). We have expanded the text by introducing a new subsection in the Supporting information section (“Sleep-like oscillatory dynamics”, line 684) aiming at illustrating that the combination of homeostatic effect and sharpening of synaptic weights induced by the network dynamics leads to an improvement in classification

performance. To do so, we made some new simulations in which we modified the asymmetry parameter and we neglected the associative effect of sleep. As shown in the inset of Figure 9 in Supporting Information, panel C, a change in the asymmetry parameter affects the homogenization effect over cortical firing rates distribution during classification phases.

For the sake of convenience, we report here the new Figure provided:

Supporting information section - “Sleep-like oscillatory dynamics”, line 712

Also, we measured the homogenization effect on cortical firing rates distributions in classification phase and related it to the asymmetry parameter. In Fig.9C we compare the network pre- and post-sleep classification performances with the firing rates homogenization effect and provide the relation between this and the asymmetry parameter in the inset.

Figure 9 - panel C:

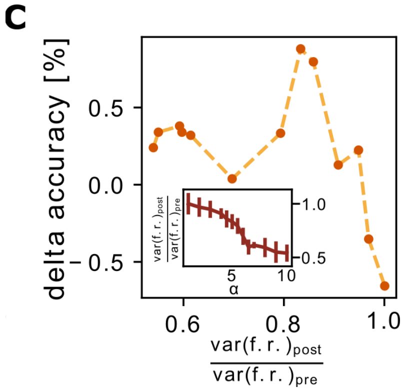

Effects of deep-sleep activity on network performances investigation. [...] **C**) We evaluated the homogenization effect due to the sleeping phase as the ratio of post- and pre- sleep firing rate variance in the classification phase. As shown in the inset, this value is handled through the change of the asymmetry parameter α.

- **R2Q4**: *I'd like to learn more about the sleep used. This is not clearly described, although delta waves are mentioned. Are there UP and DOWN states - or essentially bursts of firing between delta waves?*

We thank the reviewer for the question. We propose to add a section named “Sleep-like oscillatory dynamics” in the Supporting information section, with the text following this answer. We point to this additional section from line 203 of the main paper. Furthermore, to provide a better explanation of the network dynamics during sleep and classification, we improved Figure 3 (see figure below), which is now more informative about the dynamics occurring during sleep. In the new version, we report the variation of SO frequency across sleep (which is 1Hz at the beginning of the sleep phase and then decreases). In Figure 3, panel A, we also provide measures of input and adaptation currents, recording the average input current and adaptation current for each neuron, in different states (classification pre-sleep, early sleep, late sleep and classification post-sleep).

Line 203

When approaching the design of the ThaCo spiking model, an improvement of what some of us presented in [Capone et al. 2019], we relied on the well-established framework of Mean-Field theories [Gigante et al 2017, Capone et al 2017], to construct a network capable of spontaneously displaying two different dynamical regimes. This is obtained by acting on some parameters of the excitatory neurons (specifically, the spike frequency adaptation (SFA) and the excitatory synaptic conductance), to model acetylcholine-mediated neuromodulation on neural dynamics that supports the transitions between awake-like asynchronous activity and deep-sleep-like slow oscillations [Goldman et al 2019]. Specifically, in Fig.3A we show the incoming current to each cortical neuron versus its adaptation current during different network stages (pre- and post-sleep classification, beginning and end of the sleeping phase). In particular, sleep states are characterized by high levels of spike frequency adaptation currents (obtained through a modulation of the SFA parameter), inducing oscillations. Moreover, late sleep and after-sleep classification have low levels of input currents, due to a sleep-mediated synaptic depression leading to a reduction in the current circulating in the network.

Supporting information section - “Sleep-like oscillatory dynamics”, line 684

The rhythmic activity displayed during the sleeping phase by the network is a property that emerges when adaptation is included in a model with two stable states characterized by low and high levels of activity (Down and Up respectively). If the parameters are chosen properly, the two states become metastable, and the system regularly oscillates between the two states. Sleep states are characterized by high levels of spike frequency adaptation currents. Under this condition, the firing rate in the network exhibits spontaneous oscillations between the two fixed points. Each neuron is represented by a dot in the top panel of Fig.3 in the main text. The progressive depletion in strength of recurrent synapses induced by depressive STDP produces a reduction in the current circulating in the network, and consequently in the adaptation value. This mechanistic chain is responsible for the decrease in SO frequency reported in panel B (top). Late sleep and after-sleep classification have low levels of input currents, due to sleep-mediated synaptic depression.

New Figure 3

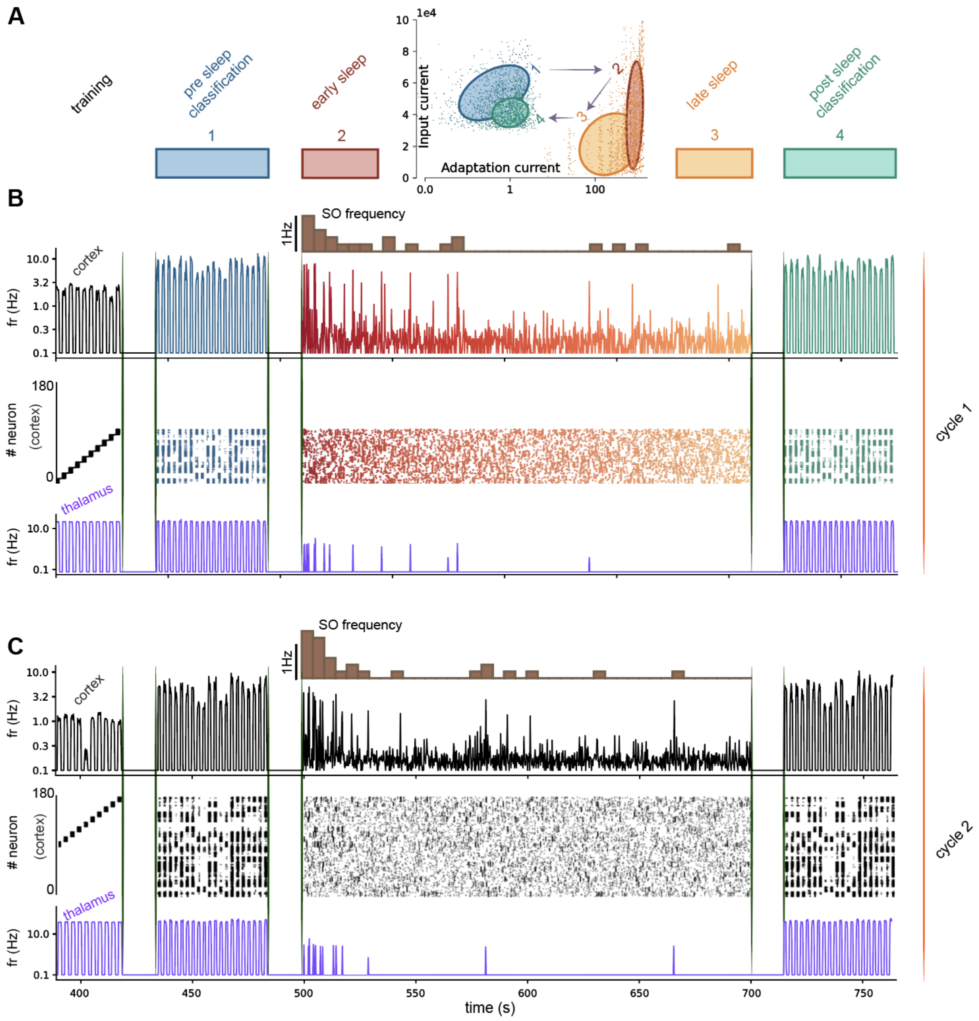

Figure 3. Incremental learning with alternation of awake training and sleep in ThaCo. **A)** *Dots:* incoming current to each cortical neuron versus its adaptation current during different network stages of the activity presented in B. Pre-sleep classification phase (represented in blue, number 1), early sleep-like phase (in red, number 2), late sleep-like phase (in orange, number 3) and post-sleep classification phase (in green, number 4). *Ellipses*: Areas in the plot associated with different network stages, estimated from data through a Gaussian Mixture Model with a full covariance matrix. During sleep, the total input current to the cortical neurons decreases due to the sleep-induced homeostatic effect, that reduces recurrent connections weights in the cortical layer (see the transition from number 2 to number 3 in the diagram), notwithstanding the constant external aspecific stimulus. Sleep-like activity, on the other hand, affects the network status during the following awake classification phase 4: the effect of STDP during sleep is a general reduction and homogenization of input current distribution, as shown in a comparison between the pre-sleep stage 1 and post-sleep stage 4 in the diagram. **B** and **C**) Spiking cortical and thalamic activity produced during training (10 examples, one per digit class), classification (20 images) and sleeping phase for two consecutive sets respectively. First row: mean firing rate of the cortical neurons trained over a set of 10 examples (each set is used to independently train 200 cortical neurons, 20 per digit example); during the sleeping phase, the slow oscillation frequency trend in time is also depicted. Second row: raster plot of the

first 400 cortical neurons. Third row: mean firing rates of thalamic neurons. Once recruited in the training phases, the cortical neurons participate in classification and sleeping phases.

- **R2Q5**: *What firing dynamics are assumed in these bursts of firing?*

The neurons we used are AdEX with no intrinsic bursting dynamics. Bursts result from a large input current inducing a high firing rate response. During training, this high level of the input current is provided by the temporal coincidence between perceptual and contextual signals impinging on the neurons. During classification, bursts are the result of the combination of the current injected by perceptual signals and of that carried by recurrent synapsis. Recurrent synapses are strengthened by the synaptic STDP dynamics as a result of the training protocol: it should be emphasized that all the cortical neurons are exposed to the thalamic stimulus, but only subgroups of them are exposed to the contextual stimulus while each given example is presented to the network. As a consequence, only some of the neurons display bursting dynamics during the presentation of each example. As a result of the training, the group will be preferentially tuned to co-activate in response to images similar to the training example. To better explain this, we added specific text to the paper, reported here:

Line 301

During the training phase, simultaneously with the input sensory-like stimulus, contextual signals are transmitted to the excitatory neurons of the cortex and to the readout neurons. The observed bursting behaviour of the neurons is a consequence of the temporal coincidence between impinging perceptual and contextual signals.

Line 325

During this [classification] stage, the neuronal activation results from the combination of the current injected by perceptual signals and the one injected by recurrent interconnections strengthened by the synaptic STDP dynamics during the training and modified by STDP during sleep cycles.

- **R2Q6**: *How might those work via STDP to achieve the synaptic downscaling shown? Or was it just the homogenization of firing rates shown in figure 2? Some of what was done may be reminiscent of Levenstein et al 2017:* *https://www.ncbi.nlm.nih.gov/pmc/articles/PMC5511069/, but I cannot tell if that is what is used here. Or really any details about the sleep paradigm. These need to be explained more clearly if the reader is to understand the experiments done.*

We thank the reviewer for having suggested this interesting work, we have cited it in the Introduction at line 68. As discussed above, homogenization is a consequence of asymmetric STDP. Indeed, there is a generalized consensus among experimentalists [Watson et al 2016; Tononi et al 2014] concerning a generalized depression of synapses during deep sleep. To emulate this effect, we set the STDP asymmetry parameter α to induce a dominance of depression (see equation in the Supporting information section at line 730). The high firing rate during up states, together with the depressive branch of STDP induce the preferential depression of stronger synapses. To better explain how firing rate homogenization and synaptic downscaling are obtained from this model and how they affect

the network classification capability, we specifically added a section “Sleep-like oscillatory dynamics” in the Supporting information section (line 684).

- **R2Q7**: *I think the basic goals, logic and setup of the training and classification need to be explained. It is not directly explained anywhere, but after much reading and confusion, I was able to understand that digit stimuli are given to the network and then classification must be a test to determine whether new stimuli are placed into the proper number category based on the criteria described around line 192. This, or whatever logic should be clearly explained. Also, it should be clearly explained that multiple stimuli are given during training, but only a single stimulus is shown during classification. Is accuracy based on the network classifying the number stimuli into the correct (ie human-based) number categories? Again, I don't think I saw any of this explained and it took me a long time to piece it together. I gather that my lack of understanding of this is based on my background in biology rather than ANNs, but explaining this basic logic will help many readers and is necessary for my review.*

Indeed, the training and classification protocol was not properly described in the previous submission. For an explanation of training and classification goals, logic and setup we added the text after this answer. Also, as the reviewer specifies, accuracy is indeed intended here as a measure of the network capability to correctly classify new unseen examples into the proper digit class (human-assigned label, provided as part of the MNIST data-set). Specifically, it is defined as the number of correctly classified examples over the total number of provided testing examples. Thus, we add some text at the beginning of the Results section (lines 153, 165) to better introduce and clarify the aims of this work. Also, we extended subsection 2.2 (line 292) in Results to better explain the mechanism underlying training and classification phases. Finally, as suggested by R2Q9, we moved the training and classification protocol figure from the Supporting information section to the main text, adding its content as new panels in Fig.7. The reviewer could also check the answer we provided to R1Q3 posed by the other reviewer.

We report below the added text to the paper:

Line 153:

In this work, we test the capability of the implemented thalamo-cortical network model (ThaCo) of expressing incremental learning when trained to learn and recall images (from the MNIST dataset), and we investigated the role and the mechanisms of the occurrence of biological-like deep-sleep dynamics.

Line 165:

The learning ability of the model was assessed using an approach that alternates incremental training with tests meant to evaluate the pre-sleep and post-sleep classification performance. During the training phase, samples are randomly extracted from the training set of the MNIST database; they are given in input to the system together with example-specific contextual signals that reach the cortical neurons, and a digit-class-specific contextual signal that reaches only the read-out neurons. Notably, to stress out the difference with respect to the training phase, during the classification phase no contextual signal is transmitted: the response of the network is recorded, based on the firing rates of the excitatory neurons of the cortex.

Line 292:

The learning protocol is organized in an alternation of training phases - when the internal structure of the network is shaped according to the learnt examples - and testing phases - when the classification performance of the network is evaluated (see Section 4.1 and in particular Fig.7A and B for more details). In both training and classification phases, the network is provided with sample images drawn from the MNIST dataset. The sample images are pre-processed to produce stimulus signals that are transmitted to the excitatory neurons of the thalamus (see Supporting Information section, paragraphs “The datasets of handwritten characters” and “Thalamic coding of visual stimuli” for more details).

During the training phase, simultaneously with the input sensory-like stimulus, contextual signals are transmitted to the excitatory neurons of the cortex and to the readout neurons. The observed bursting behaviour of the neurons is a consequence of the temporal coincidence between impinging perceptual and contextual signals. Specifically, for each example to learn, an example-specific group of excitatory neurons in *cx* is facilitated through the presentation of a contextual signal. This induces a higher activity in these neurons causing a strengthening of both thalamo-cortical synapses and recurrent synapses. This example-specific tuning involves each neuron with a single training example only, whose category defines (in an unsupervised manner) a natural category for which the neuron is better tuned. Meanwhile, a subgroup of readout neurons (*ro*) is stimulated by a digit-class specific contextual signal, leading to an enhancement of connections between the cortical neurons trained over the presented example and the subgroup of readout neurons associated with the correct class. [...].

During the classification phase, signals resulting from preprocessed images are again transmitted to the thalamus analogously to the training phase, however, no contextual signal is transmitted to either cortical and readout neurons. During this stage, the neuronal activation results from the combination of the current injected by perceptual signals and the one injected by recurrent interconnections strengthened by the synaptic STDP dynamics during the training and modified by STDP during sleep cycles.

- **R2Q8**. *I still do not understand the experimental set-up implied in the “synapses trained over the same example” and other elements of figure 5B. I am afraid I still do not understand the fundamentals of the work here, since they were not explicitly explained. I was surprised to see that perhaps not all neurons in the network were exposed to all examples. Am I correct in understanding that? If not, can the authors better explain the training paradigm and how the different groups in 5B are generated. I think training needs to be fully explained, as does classification measurement. In Figure 6, how does a “neuron belong to a different category” (as stated in Fig 6 legend)? Does that mean it responds maximally to a different stimulus? Or was trained on only one stimulus? Again, the details of training are not explained, making the paper difficult to understand.*

We thank the reviewer for the question; we improved the caption of figure 5B and we added text to describe the training protocol.

It should be emphasized that all the cortical neurons are exposed to the thalamic stimulus for all the training examples (training phase) and all the images to be classified (classification phase).

While training the network on one example, all the cortical neurons receive the sensory input from the thalamus (coding for the visual feature of the digit, see Fig. 7C.a). However, only a group of neurons receive the contextual signal coding for that specific example. The simultaneous arrival of perceptual and contextual signals on cortical neurons induces the bursting dynamics (Fig. 7C.c), but those reached by the example specific contextual signal better tune on that specific thalamic pattern. For simplicity, we report Fig7 below.

During the presentation time of each training example, a subset of cortical neurons (20 in this work) is reached by a contextual signal in addition to the thalamic stimulus. This induces a higher activity in those neurons producing both a strengthening of thalamo-cortical synapses and recurrent synapses. This example-specific tuning happens for each neuron only on a single example. The category of that example defines (in an unsupervised manner) a natural category for which the neuron is better tuned.

During the classification phase, the temporal coincidence of the current carried by recurrent cortical synapses (strengthened during training) and the sensory input from the thalamus induces the bursting in those groups of neurons for which there is a higher similarity between the thalamic stimulus and examples received during the training.

One way to perform classification is to observe the category of the neuron maximally responding to a new presented example. This has been deeply rephrased in the main text, shown in the previous answer. We also modified the caption of Figures 5 and 6 introducing the phrase: “group of neurons simultaneously reached by a higher value of the contextual signal”.

Line 305:

Specifically, for each example to learn, an example-specific group of excitatory neurons in *cx* is facilitated through the presentation of a contextual signal. This induces a higher activity in these neurons causing a strengthening of both thalamo-cortical synapses and recurrent synapses. This example-specific tuning involves each neuron with a single training example only, whose category defines (in an unsupervised manner) a natural category for which the neuron is better tuned.

Caption fig. 5:

A) **Sleep mitigates the effects of noisy contextual signals on the classification performances of ThaCo.** Classification performances evaluated over the Readout layer (supervised learning protocol, left) and Cortical layer (unsupervised learning protocol, right). Comparison among the network trained over non-noisy examples (blue), the network trained over noisy examples without any deep-sleep like phase (violet), and the network trained over noisy examples interposing a deep-sleep-like activity between the training and the classification phases (green). The contextual signal provided in the training phase is corrupted by noise (i.e. some examples are associated with stronger synapses), leading to a drop in performances (comparison between blue and violet line). Still, the interposition deep-sleep-like phases between noisy-training and classification phases recover the

performances of the network trained with a non-noisy protocol. B) Sleep-induced homeostatic and associative effects on cortico-cortical synaptic-weight distributions. Pre-sleep (violet), post-sleep (green). Solid lines: mean and standard deviation. a) Intra-group connections: weight distributions of synapses connecting neurons trained over the same example (i.e. that during the training stage were triggered by the same contextual stimulus, thus activated simultaneously during a specific training example); b) Intra-class connections: weight distributions of synapses connecting neurons trained over different examples belonging to the same class (i.e. i.e. that have not been simultaneously triggered by the contextual stimulus in the training phase, but still have been triggered by a sensorial thalamic signal associated to images belonging to the same class); c) Inter-class connections: Connections among groups trained over different classes (i.e. i.e. triggered by the contextual stimulus together with a sensorial thalamic signal associated to images belonging to different classes). We note the homeostatic effect of sleep (in A) leading to a general reduction of weights associated with example specific synapses and a reinforcement of the intra-class connections (in B). Synapses connecting groups trained on different examples, on the other hand, are much less affected by sleep. The inset, showing part of the same plot in a lin-lin scale, is added to illustrate the shape of the pre-sleep distribution, and the difference in the mean values before and after sleep.

Caption fig. 6:

[...] A) and B) depict all cortico-cortical synaptic weights connecting the full set of trained neurons (colour bar, logarithm scale), black lines separate neurons solicited by contextual signal together with thalamic sensorial signal pointing to images belonging to the same digit class in the training phase; C) and D)focus on the synaptic weights connecting groups of cortical neurons simultaneously solicited by a contextual signal in the training phase (thus stimulated over the same sensorial signal identifying images belonging to the same class) (colour bar, linear scale), vertical black lines separate neurons trained over different categories, horizontal black lines separate cortical groups of neurons solicited during the presentation of 10 examples (one per digit class). [...]

- **R2Q9**: Figures S1 should be brought early into the paper as part of an explanation of the basic experiment as mentioned above.

We moved Figure S1 - illustrating the training and classification protocol- from the Supporting information section to the main text, adding its content as new panels A and B in Figure 7. We point it from section 2.2 at line 334. The text added as an answer to questions R2Q7, R2Q8 should now provide a complete description of the training and classification protocol and methods.

MINOR ISSUES:

- I cannot tell if “ro” as in figure 1 is defined anywhere. I assume it stand for “readout”

Yes indeed. We added some text in the caption of Figure 1 to define it.

- Line 62; “higher rate” maybe should be “highest rate”

As depicted in Figure 7 panels C and D, during the training phase, a hard WTA dynamics is implemented: only the group of cortical neurons associated with the highest firing rates survives whereas the others are suppressed. During the classification phase, on the other hand, soft WTA dynamics is implemented: even if the low firing groups are suppressed by inhibition, the high-firing groups survive (not necessarily only the highest). We better explained this by modifying line 60 in the revised document as follows:

Within appropriate conditions, the inhibitory signal will be sufficiently high to suppress the signal of all the low-firing excitatory groups of neurons whereas the high-firing ones survive.

- Line 136: “mice” should be “rats”

Yes indeed. We thank the reviewer for having pointed this error out. We modified the text coherently.
